# Bone Regeneration Guided by a Magnetized Scaffold in an Ovine Defect Model

**DOI:** 10.3390/ijms24010747

**Published:** 2023-01-01

**Authors:** Melania Maglio, Maria Sartori, Alessandro Gambardella, Tatiana Shelyakova, Valentin Alek Dediu, Matteo Santin, Yolanda Piñeiro, Manuel Bañobre López, Josè Rivas, Anna Tampieri, Simone Sprio, Lucia Martini, Alessandro Gatti, Alessandro Russo, Gianluca Giavaresi, Milena Fini

**Affiliations:** 1 Surgical Sciences and Technologies, IRCCS Istituto Ortopedico Rizzoli, 40136 Bologna, Italy; 2 Istituto per lo Studio dei Materiali Nanostrutturati, Consiglio Nazionale delle Ricerche, 40129 Bologna, Italy; 3 Centre for Regenerative Medicine and Devices, School of Applied Sciences, University of Brighton Huxley Building Lewes Road, Brighton BN2 4GJ, UK; 4 Department of Applied Physics, University of Santiago de Compostela, E15782 Santiago de Compostela, Spain; 5 INL—International Iberian Nanotechnology Laboratory, 4715-330 Braga, Portugal; 6 Institute of Science, Technology and Sustainability for Ceramics-National Research Council (ISSMC-CNR, Former ISTEC), 48018 Faenza, Italy; 7 II Clinic of Orthopaedics and Traumatology, IRCCS Istituto Ortopedico Rizzoli, 40136 Bologna, Italy

**Keywords:** magnetic scaffold, nanoparticles, VEGF, critical size defect, ovine model, histomorphometry, AFM

## Abstract

The reconstruction of large segmental defects still represents a critical issue in the orthopedic field. The use of functionalized scaffolds able to create a magnetic environment is a fascinating option to guide the onset of regenerative processes. In the present study, a porous hydroxyapatite scaffold, incorporating superparamagnetic Fe_3_O_4_ nanoparticles (MNPs), was implanted in a critical bone defect realized in sheep metatarsus. Superparamagnetic nanoparticles functionalized with hyperbranched poly(epsilon-Lysine) peptides and physically complexed with vascular endothelial growth factor (VEGF) where injected in situ to penetrate the magnetic scaffold. The scaffold was fixed with cylindrical permanent NdFeB magnets implanted proximally, and the magnetic forces generated by the magnets enabled the capture of the injected nanoparticles forming a VEGF gradient in its porosity. After 16 weeks, histomorphometric measurements were performed to quantify bone growth and bone-to-implant contact, while the mechanical properties of regenerated bone via an atomic force microscopy (AFM) analysis were investigated. The results showed increased bone regeneration at the magnetized interface; this regeneration was higher in the VEGF-MNP-treated group, while the nanomechanical behavior of the tissue was similar to the pattern of the magnetic field distribution. This new approach provides insights into the ability of magnetic technologies to stimulate bone formation, improving bone/scaffold interaction.

## 1. Introduction

Critical bone loss is a frequent consequence of many pathological conditions such as trauma, oncologic diseases and infection processes, that may frequently require invasive treatments to be managed. The repair of critical bone defects is a challenging and time- requiring process, which can be delayed or impaired by several factors beyond the size of the defect and the anatomical location [1,2]. Ageing, unhealthy lifestyle and previous or concomitant pathologies can affect the healing process, leading to delayed union or nonunion [3].

Among the various aspects competing to the fracture healing, an adequate vascular supply is essential [4]. In an initial step, revascularization competes in the injured tissue debris and microorganisms’ removal, in association with phagocytes, but also allows the recruitment of mesenchymal osteochondro-progenitor cells, necessary for the regeneration of the tissue. An impairment in this key step can lead to incomplete or inefficient healing, which compromises the biological characteristics and mechanical competence of the newly formed bone [5]. Consequently, the ideal approach for the management of a critical size bone defect is to create the adequate microenvironment able to promote the onset of a successful healing process. For this purpose, the presence of osteoinductive and angiogenic factors is necessary, namely growth factors such as transforming growth factor-beta (TGF-beta), VEGFs and proinflammatory cytokines, delivered to the site of injury by vasculature, promoting the differentiations of mesenchymal stem cells to osteoblasts. In addition, an osteoconductive structure promotes cell activity and the consequent bone apposition and growth [6]. In the latest years, many tissue engineering-based strategies have been employed for therapeutic approaches mixing the use of scaffolds/biomaterials with biological adjuvant as cells (e.g., mesenchymal stem cells, embryonic stem cells, etc.) and/or growth factors, such as bone morphogenetic proteins (BMPs), vascular endothelial growth factors (VEGFs), transforming growth factor (TGF-beta1) or mechanical stimulation [7,8].

The growing use of superparamagnetic nanoparticles (MNPs) in versatile medical applications, such as gene delivery, hyperthermia treatment of tumors, magnetic resonance imaging (MRI) and magnetic cells separation, has pushed the interest towards the application of the principles of magnetism and the use of magnetic materials for promoting bone regeneration [9].

In this respect, it is widely accepted that MNPs can exert a trophic effect on cells, stimulating proliferation and differentiation towards osteoblastic lineage [10,11]. These properties can be further enhanced if MNPs are combined with scaffolds, hence, allowing the generation of a magnetic environment; such strengthening can have a significant impact on the mechanical behavior of the regenerated bone, thanks to the capability of the scaffold to improve cell adhesion and, as a consequence, the mechanical properties of the tissue [12].

Several groups have shown that MNPs can be easily guided in vivo; however, the use of an internal device capable of operating on a local scale is crucial to improve their placement and reduce dispersion phenomena [13].

It is straightforward that the attainment of high mechanical stability at the interface, with a consequent reduction in micromovements, is critical to improving the regenerative process [14,15].

The aim of the present study was to evaluate the regenerative potential of a specifically designed magnetic ceramic scaffold in an ovine model of critical size defect. The scaffold design was optimized to exert the regenerative action via an in situ injection of MNPs conjugated with VEGF through docking sites obtained by functionalization with hyperbranched poly(epsilon-Lysine) dendrimer. The VEGF-loaded MNPs were transported throughout the scaffold porosity to generate a VEGF gradient able to stimulate angiogenesis in the regenerating bone.

## 2. Results

### 2.1. Magnetic Simulations

**Figure 1:**
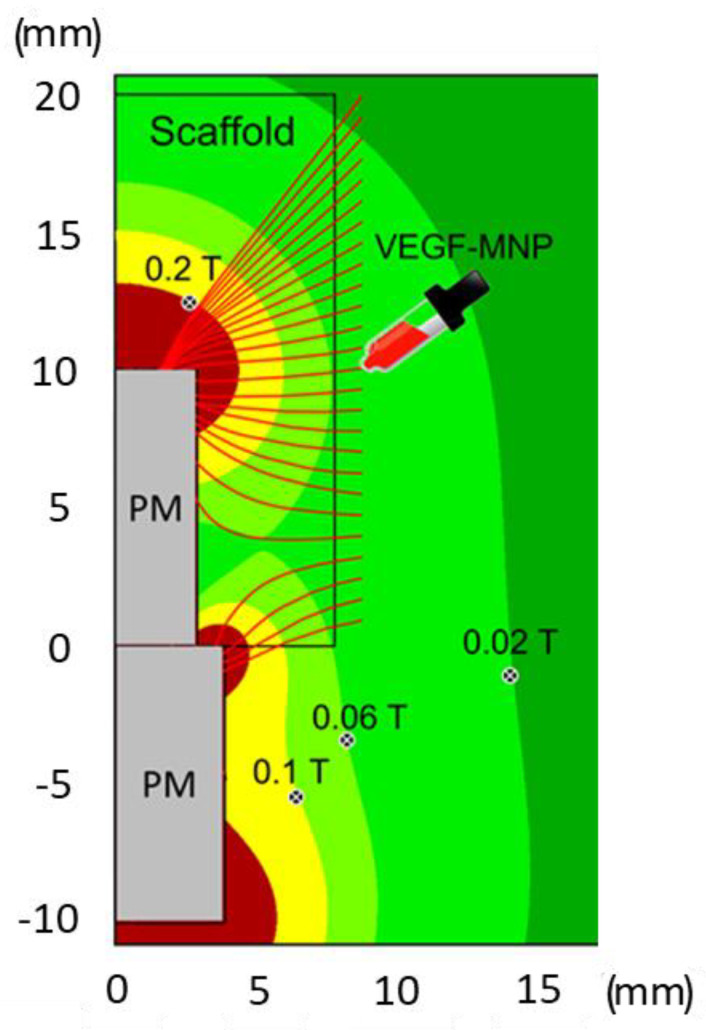
Trajectories (red lines) of VEGF-MNPs injected near the scaffold surface. The color distribution shows isolines of the magnetic field (B = 0.02–0.2 T) near the scaffold and permanent magnets (PMs).

Figure 1 shows a radial section of the magnetic field distribution near the permanent magnets (PMs) and in the scaffold zone. It evidences isolines with magnetic field values equal to 0.02, 0.06, 0.1 and 0.2 T. The magnetic field B generated in this configuration reaches its maximum Bmax ≈ 1 T near the ends (brown zones). The red lines represent trajectories of motion of VEGF-MNP complexes distributed initially near the scaffold surface. Indeed, the VEGF-MNP injection is positioned in the middle of the scaffold (Figure 1), exploiting the available space between the scaffold and tissue produced by the surgery. After the injection, the growth factors moved under the effect of the magnetic field inside the scaffold following the trajectories indicated. This led to a VEGF distributed in the entire scaffold. The simulations show that at the investigated scaffold magnetization there was no influence of the scaffold on the VEGF trajectories; they were fully determined by the two permanent magnets. However, magnetic inhomogeneities, such as MNP agglomerates may have attracted and trapped magnetized VEGF.

**Figure 2:**
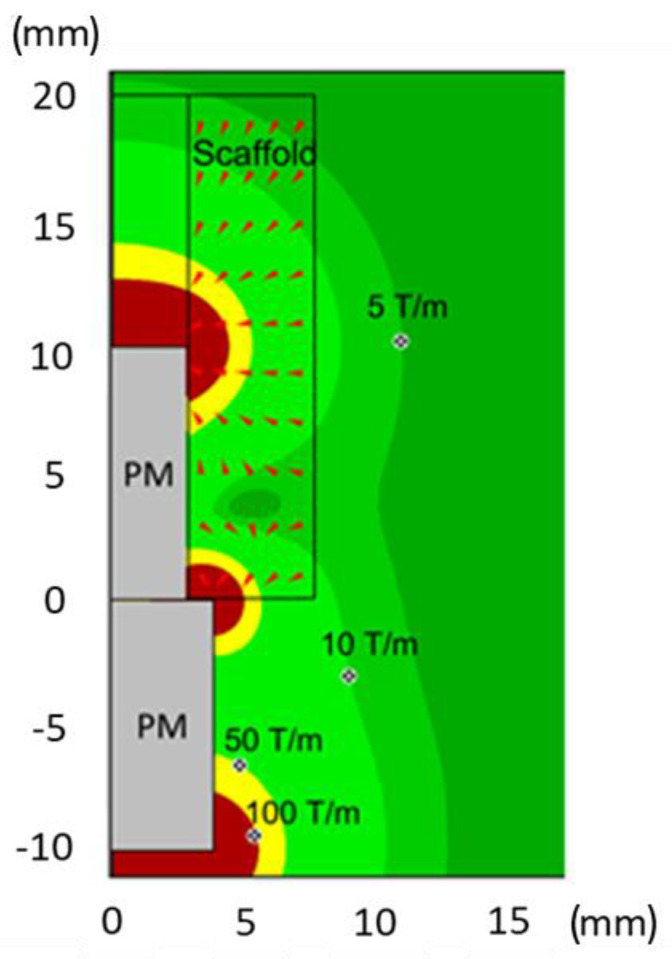
Directions of magnetic forces acting on MNPs dispersed inside the scaffold. The color distribution shows isolines of the magnetic field gradient, which grows from dark green to brown.

Next, we modeled the behavior of magnetic nanoparticles employed to magnetize the scaffolds. At a given time after the scaffold implantation, the scaffold structure initiates to “dissolve”. The acting magnetic forces are proportional to the magnetic field gradient. Figure 2 shows isolines of the magnetic field gradient equal to 5, 10, 50 and 100 T/m and the arrows show the direction of the magnetic forces acting on the magnetic component of the scaffold (radial section). Close to the magnet edges the gradient reaches enormous values of a few thousand T/m. The regions of maximal values of the magnetic field (Figure 1) and magnetic gradients correspond effectively to the regions of maximal magnetic forces acting on the MNPs.

**Figure 3:**
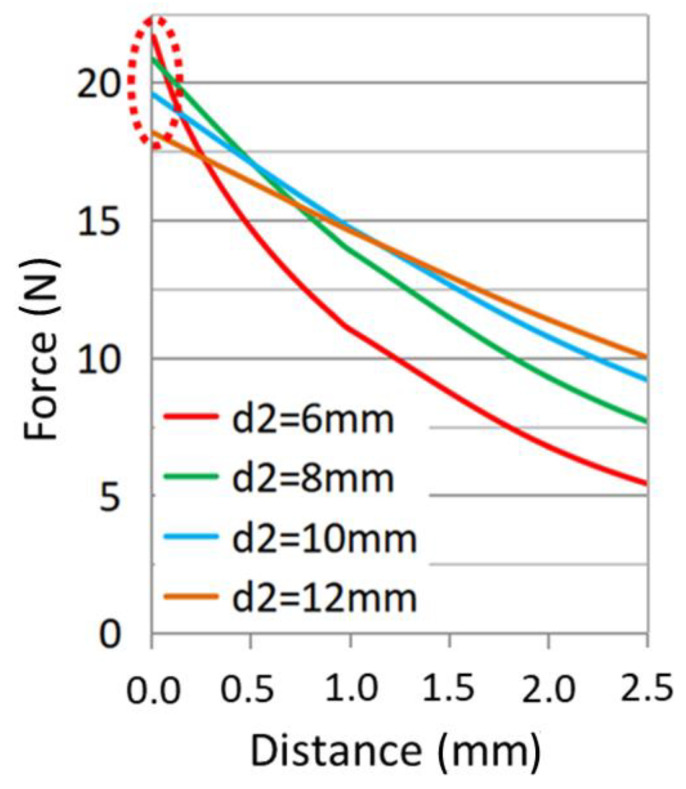
Attraction forces between two permanent magnets having height 10 mm and diameters d1 = 6 mm and d2 = 6–12 mm versus distance between them.

The force between the two permanent magnets (one fixed inside the scaffold and the other one in the bone medullar canal), allowed for an additional magnetic fixation and the reduction in micromotions at the interface between the scaffold and tissue. Figure 3 displays the attractive force between the chosen permanent magnets; the size of the magnet fitted into the scaffold was constant (d1 = 6 mm, h = 10 mm), while the diameters of the other magnets were defined by bone medullar canals. It was seen that the attraction forces could reach up to 20 N for short distances.

### 2.2. In Vivo Study

At the time of harvesting, the macroscopic observation did not highlight evidence of an inflammatory reaction at the site of implantation, neither a sign of hematoma nor oedema.

**Figure 4:**
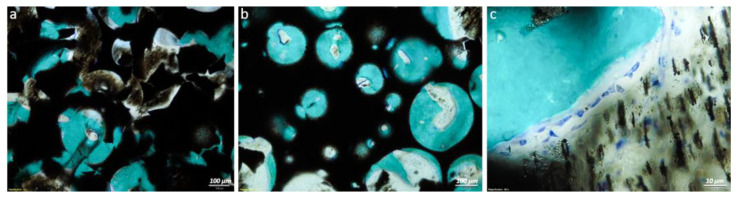
Histological images of resin-embedded samples from the magnetic scaffold + VEGF-MNPs treated group (**a**,**c**) and magnetic scaffold group (**b**) highlighting the growth of new bone filling the porosity of the scaffold and the regions in contact with it (**a**,**b**) and the presence of osteoblasts along bone trabeculae (**c**). Toluidine blue/fast green staining with 8× magnification—100 μm scale bar (**a**,**b**) and 80× magnification—10 μm scale bar (**c**).

At histological observation, in both experimental groups it was possible to appreciate the growth of bone tissue in direct contact with the materials. In particular, the growth of bone tissue was observed along the entire length and width of the implant, with the porosity of the scaffold filled with newly formed bone. Fast green histological staining made it possible to appreciate the presence of new bone which had a more intense and brilliant stain than the pre-existing bone at the extremities. In some small areas where bone regeneration was not yet complete there was connective tissue. The presence of cells and chains of osteoblasts along the bone trabeculae was appreciated (Figure 4).

**Figure 5:**
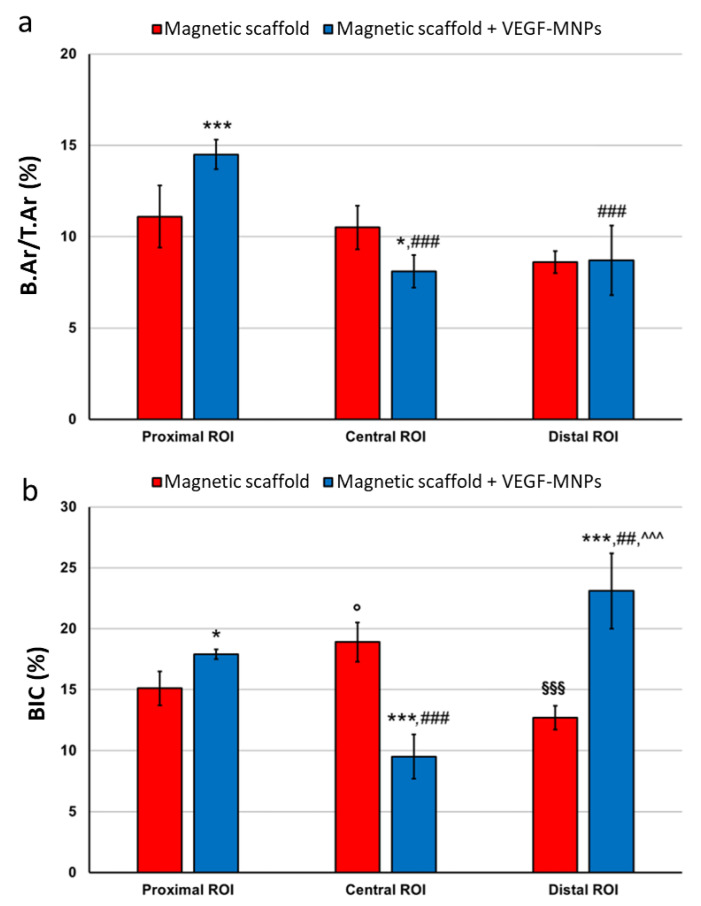
Bars of bone area/total area (%) (B.Ar/T.Ar) and BIC (%) of magnetic scaffold + VEGF-MNPs group and magnetic scaffold group measured in the proximal, central and distal ROIs. Two-way ANOVA showed significant interactions of selected factors (treatment and ROI) on B.Ar/T.Ar (F = 9.40, *p* = 0.002) and BIC (F = 29.09, *p* < 0.0005). Pairwise comparison test (1 symbol: *p* < 0.05; 2 symbols: *p* < 0.005; 3 symbols: *p* < 0.0005); *, magnetic scaffold + VEGF-MNPs group versus magnetic scaffold group; °, central and distal ROIs versus proximal ROI for magnetic scaffold group; #, central and distal ROIs versus proximal ROI for magnetic scaffold + VEGF-MNPs group; §, distal ROI versus central ROI for magnetic scaffold group; ^, distal ROI versus central ROI for magnetic scaffold + VEGF-MNPs group.

A histomorphometric analysis showed trends along the regions of interest (ROIs) identified starting from the magnet. Statistically significant differences were observed in bone area (B.Ar) between the two experimental groups in the proximal ROI (*p* < 0.0005) and in the central ROI (*p* = 0.02), indicating a greater bone area in the samples treated with VEGF in the areas in greater proximity to the magnet. Bone-to-implant contact (BIC) values showed greater contact in the VEGF group in the proximal (*p* = 0.037) and distal (*p* < 0.0005) ROIs and an inverted trend in the central ROI (*p* < 0.0005) (Figure 5).

### 2.3. AFM Nanoindentation

**Figure 6:**
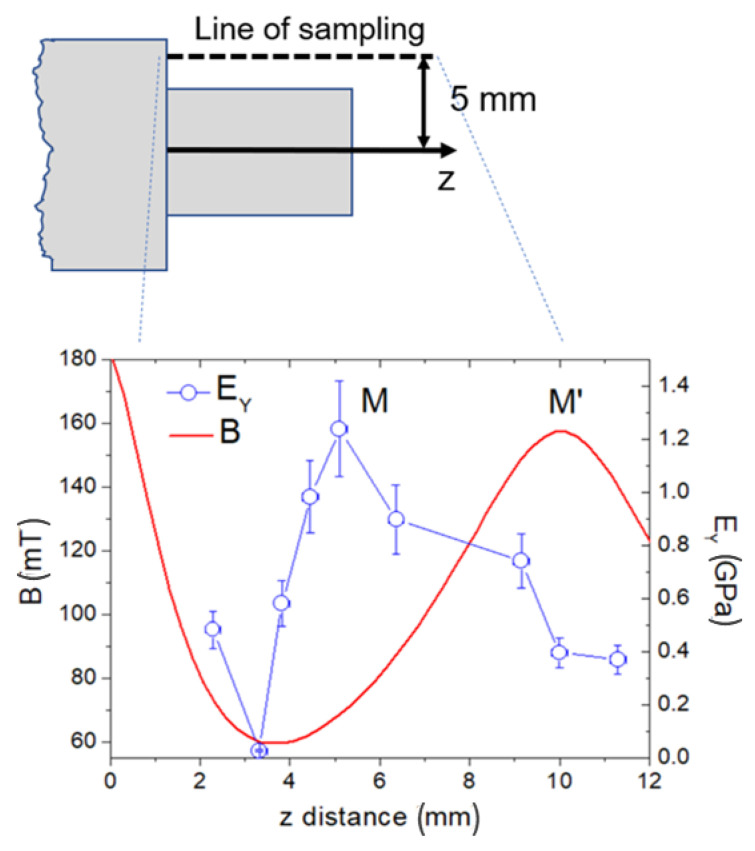
Graph of the Young’s modulus (blue curve) estimated via AFM nanoindentation and the field B (red curve), both taken along a straight line at a fixed (5 mm) distance from the *z*-axis.

In Figure 6, the variation in the Young’s modulus (E values) along the direction parallel to the *z*-axis is shown together with the field values (B values) at the same distance from the *z*-axis. Remarkably, there is a variation in E along the *z*-axis with a minimum m = (0.025 ± 0.004) GPa at z = (3.32 ± 0.17) mm and a maximum M = (1.24 ± 0.18) GPa at z = (5.10 ± 0.17) mm; furthermore, a gradual decrease at higher z values was observed.

## 3. Discussion

VEGF plays a pivotal role in coupling angiogenic and osteogenic processes. This peculiar ability makes VEGF a key element in bone tissue engineering strategies, especially aimed at the repair of critical size defects. A huge variety of approaches for biological reconstruction have been tested and implemented over the years to overcome the use of prosthetic devices, exploiting the use of bone cement spacers and induced-membrane techniques in viable and nonviable bone grafts, with different preparations and attempts to promote vascularization [16].

Current strategies described in the literature foresee the use of angiogenic and osteogenic growth factors, stem cells, genes or a combination of these in addition to biomaterials/scaffolds, with appropriate characteristics of porosity and interconnectivity, to enhance the intrinsic abilities of all players to stimulate bone regeneration, exploiting the possible mechanical stability provided by the scaffold, promoting the spreading of a vascular network. In many cases, the scaffolds are designed to support a targeted delivery of the biological components in order to find the optimal release and action times to promote the biological phenomena underlying bone healing [17].

MNPs are a common vehicle for the delivery and/or release of biological agents controlled with magnetic fields. In addition, they present a further advantage by improving the stability of the carried bioactive agent and inducing the formation of a concentration gradient mimicking the structure of the target tissue. In bone tissue engineering, MNPs can be incorporated as part of the scaffold, enhancing osteogenesis via cell proliferation and differentiation, and acting on the mechanical stability with trends that seem to correlate with MNP concentration [12,18].

Previous studies of the present group have focused on the development and characterization of the magnetic scaffold for in vivo guided bone growth.

The biocompatibility and osteoconductive properties of magnetic hybrid scaffolds (HA/magnetite) in comparison to pure HA scaffolds have been previously investigated in vitro and in a rabbit model of bone regeneration up to 12 weeks, highlighting high bone formation and a maintenance of the tissue’s mechanical properties [19]. In an analogue animal model, the synergic effect of a hybrid collagen/HA scaffold magnetized with MNPs and a titanium-coated permanent magnet was investigated. Results showed a synergic action of the scaffold and magnetic fields with a peculiar effect on the collagen fibers network organization, indicating an alignment of the fibers in the same direction of the magnetic field, thus, suggesting a biological effect directly guided by the magnetism [20].

The use of permanent magnets and magnetic materials for bone fixation has already been discussed previously [21]. Scaffold fixation for long bone defects is generally a challenging issue and intramedullary rods are often used to align and stabilize fractures and scaffold grafts. The use of magnetic rods can improve the stability of long bone scaffolds and reduce the micromotions at the bone/scaffold interface, which is of great relevance to favor new bone formation and osteointegration.

The results of this study confirm that even though the magnetic fixation cannot fully substitute the traditional one, it nevertheless offers a significant additional support, enhancing the regeneration efficiency. In addition, the forces between two permanent magnets in the fracture model can reach considerable values, especially for long bones with large medullary canals.

Interestingly, the histomorphometric results obtained in the group undergoing VEGF-MNP administration presented a trend matching that identified with magnetic simulation, thus, supporting the evidence of previous studies about the creation of a magnetic gradient, influenced by a magnetic field, guiding bone regeneration. In fact, the effect of the VEGF administration was particularly strong in the area immediately adjacent to the permanent magnet and the interface between the scaffold and the magnet. This effect was reflected both in the area of newly formed bone and in the percentage of contact between the bone and the material, thus, indicating a greater presence of tissue in the porosity of the scaffold.

An evaluation on a local scale of the tissue biomechanics via nanoindentation is crucial to investigate the role of the magnetic field in bone tissue regeneration. In this respect, AFM, due to its high spatial resolution (at limit a few nm) and capability to detect the materials’ response to small (at limit below nN) forces applied, represents the ideal tool to measure the response of the bone with respect to an external stimulus. To do this, the stiffness of the peri-implant bone tissue was measured as a signature of bone regeneration, showing a trend similar to those described for magnetic simulation and histomorphometry, thus, confirming that the tissue formed in the area near the magnet had characteristics of stiffness similar to native bone, suggesting the onset of a successful regeneration process. In a scenario where the process of bone regeneration—intended as improved bone density as well as stiffness—is driven by MNPs, it is straightforward that these tend to align their position along the maxima of B. Thus, it is likely that the displacement observed is caused by the earlier extraction of the implant. Notably, the positions of the local minimum and maximum, m and M, are in agreement with the corresponding isolines of Figure 1 and Figure 2.

Despite the increasing availability of preclinical in vivo models for the study of innovative approaches in critical size defect healing, the use of large models, in particular ovine, still remains the most suitable choice, especially for long bone defects. In fact, ovine share important similarities with human bone characteristics such as the micro- and macroarchitecture, bone mineral compositions, mechanical features and remodeling abilities. In addition, the use of such a large animal model allows the performance of surgical procedures more similar to the clinical practice, using internal and external fixation employed for humans. All together, these aspects promise a better translation of the results obtained [22].

However, some aspects deserve further insights to enrich the study and clarify the mechanisms underlying the observed phenomena, in particular, what the optimal dose and timing of VEGF administration is and what the real interaction between the MNPs/scaffold/magnets and the involved forces is. In addition, the use of an AFM analysis to detect mechanical characteristics of tissue in the presence of a porous metallic implant with interspersed MNPs should be implemented to validate the protocol and avoid possible biases related to material debris. Eventually, future in vivo studies might be implemented with a further experimental group to make a comparison with the onset of the bone regenerative process in the presence of only traditional scaffolds.

## 4. Materials and Methods

### 4.1. Scaffold and VEGF-Conjugated MNP Synthesis and Characterization

The magnetic configuration consisted of two coaxial cylindrical permanent neodymium (NdFeB) magnets with diameters ranging from 6 to 12 mm, height of 10 mm and residual magnetization of 1.45 T (Alga Magneti srl, Italy). For biocompatibility reasons, the magnets were coated with a 7 µm parylene layer.

The magnetic scaffolds were obtained using a previously described method, starting from commercial hydroxyapatite (HA) and magnetite powders (Sigma Aldrich, Milan, Italy) [23]. Briefly, the HA powder, pretreated at 1000 °C for 5 h, was homogenized with magnetite nanoparticles (diameter <50 nm) in a 90:10 *w*/*w* ratio. Aqueous slurries were then prepared with such a powder mixture, adding 1.5 wt% of Dolapix CA (Zschimmer & Schwarz, Lahnstein, Germany) as a dispersant and 1.4 wt% of Dermocin BS Conc (Fratelli Ricci, Italia) as a foaming agent, suitable to induce the formation of air bubbles into the slurry upon 8 h of milling. Finally, the foamed suspensions were casted in paper molds, dried at room temperature for 48 h and finally sintered at 1200 °C for 1 h under an Ar/H2 atmosphere, to prevent the degradation of the magnetite into nonmagnetic iron oxides. The scaffolds exhibited a magnetization of 53 emu/g and featured a hollow geometry with a 16 mm external diameter, 6 mm internal diameter and 20 mm height.

A 6 mm diameter magnet was tightly inserted from one edge of the scaffold into its hollow volume. A corresponding magnet of 8 mm diameter was fixed into the bone providing a magnetic fixation of the scaffold in addition to the standard fixation.

The preparation, functionalization and characterization of the MNPs were previously described in detail in [24]. In brief, superparamagnetic nanoparticles coated with a silica layer were functionalized with hyperbranched poly(epsilon-Lysine) presenting 16 molecular branches, each exposing a molecule of the modified amino acid carboxybetaine. The synthesis of the hyperbranched peptides and their covalent grafting onto the silica surface of the MNPs was performed as reported in the same work. The functionalized MNPs were then conjugated with VEGF by physical interaction with the carboxybetaine through an optimized method yielding a VEGF load of 3.64 µg/mg of MNPs. The successful MNP functionalization with the hyperbranched peptides was assessed using a fast Fourier transform infrared analysis, while the quantification of their VEGF cargo was measured using the VEGF ELISA kit, as well as the release of VEGF under magnetic stimulation [24].

### 4.2. Magnetic Simulations

Computer modeling with COMSOL 3.5 (Comsol Inc., Stockholm, Sweden) was employed to calculate the magnetic characteristics for the used magnet configuration (two cylindrical permanent magnets with diameters equal to 6 and 8 mm) using the Multiphysics–Magnetostatics–2D axial symmetry application mode.

### 4.3. In Vivo Studies

In vivo studies were performed in compliance with the Italian laws on animal experimentation, upon approval by the Ethical Committee of the Rizzoli Orthopaedic Institute and by the Ministry of Health, according to Legislative Decree No. 116/92, in force at the time of submission of the protocol.

Eight mestizo adult sheep (breeding: Pancaldi Raffaele, Budrio, Bologna, Italy), with 50 ± 8 kg body weight, were housed in individual cages under controlled conditions and fed with a standard diet and water ad libitum. After a quarantine period of 5 days, the animals underwent surgery for the creation of a critical size defect in the metatarsus. The animals were premedicated with an intramuscular injection of 10 mg/kg ketamine (Imalgene 1000, Merial Italia SpA, Assago, Milan, Italy) and 0.2 mg/kg xylazine (Rompun, Bayer SpA, Milano, Italy), and a subcutaneous injection of 0.0125 mg/Kg atropine sulfate. Then, an endovenous injection of 10 mg/Kg thiopental sodium (Farmotal 2.5%, Pharmacia & Upjohn SpA, Milan, Italy) was performed to induce general anesthesia. During surgery, anesthesia was maintained with an O2/air (60/40 l/min) mixture and 1.5–2% sevorane (Sevorane, ABBOTT Srl, Latina, Italy). Upon awakening, 62.5 mL/mg of methylprednisolone sodium succinate (Solu-Medrol, Pharmacia & Upjohn SpA, Milan, Italy) was administered intravenously.

Postoperatively, antibiotics and analgesic therapies were administered and the animals were not restricted in their movements. At the end of the experimental time of 16 weeks, all animals underwent euthanasia under general anesthesia with the intravenous administration of 10 mL of Tanax (Tanax, Hoechst, Frankfurt am Main, Germany).

### 4.4. Surgical Procedure

**Figure 7:**
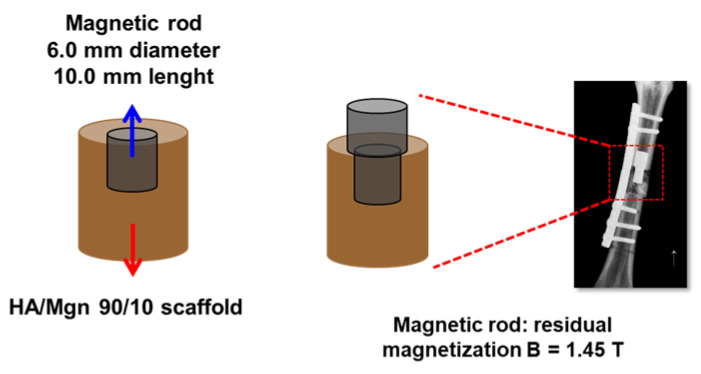
Schematic representation of the magnetic scaffold, fixed additionally with two permanent magnetic rods implanted in the critical size bone defect in vivo, and the radiographic image of the metatarsus with the positioning of the scaffold.

Surgeries were performed in aseptic conditions. Taking the animal placed on the right side, the metatarsus shaft of the right hind limb was exposed through a medial approach directly above the bone to reach the medial side. A cortical periosteal bone defect of the metatarsus of 2 cm in length was created. The diaphyseal segment was replaced with a magnetic porous ceramic scaffold of 6 mm in diameter and 2 cm in length fixed with an eight-hole plate proximally with 2 screws and distally with 3 screws. The medullary canal was reamed until it had an 8.0 mm inner diameter to allow for the cylindrical magnetic rod implant (6 mm diameter, 10 mm length). The simultaneous presence of a permanent magnet inside the scaffold and inside the host bone resulted in a compression force at the bone–scaffold interface of approximately 25 N, such as to improve the fixation of the scaffold itself. At the end of the surgical procedure, a limb cast (scotch/soft cast, 3M Health Care, 3M Italy, Milan, Italy) was applied including the limb up to the ankle joint. During these weeks, the animals were hanged by means of a suspension sling with the operated leg barley touching the ground, thus, avoiding full weight loading on the operated leg (approximately 30%) and preventing torsional or shear forces through the immobilization of the tibia shaft with the cast (Figure 7).

One week after surgery, animals were divided into two groups:

- Magnetic scaffold group (n = 4).
- Magnetic scaffold + VEGF-MNPs group (n = 4): in deep sedation, animals received a localized injection of 0.75 mL VEGF-conjugated MNPs at the intervention site.

Radiographic investigations using the Nessy HF 30/4 radiological instrument (ACEM SpA, Bologna, Italy) and the Kodak Point of Care CR system (Carestream Health Inc., Rochester, MN, USA) to digitalize radiographs were conducted to assess the positioning of the implants.

### 4.5. Histological and Histomorphometrical Assessments

At euthanasia, after macroscopic observation, bone samples were removed and immediately fixed in paraformaldehyde (Sigma Aldrich, Saint Louis, MO, USA) buffered at 4% for 48 h. After dehydration in increasing concentrations of alcohol solutions (24–48 h/each concentration), samples were embedded in polymethylmethacrylate (PMMA) (Methacrylate, Merck, Schuchardt, Hohenbrunn, Germany). After polymerization, the samples retrieved from all the animals of both groups were cut according to a frontal cutting plan (EXAKT Cutting and Grinding Systems, Apparatus GmbH & Co., Norderstedt, Germany), thinned and polished up to an 80 ± 10 μm thickness. The histological slides were stained with toluidine blue (Carlo Erba Reagents Srl, Milan, Italy) and fast green (Histoline, Milan, Italy), and representative images from each group were digitally acquired (Aperio Scanscope CS System, Aperio Technologies, Vista, CA, USA) at maximum resolution (1781 × 1467 pixels).

**Figure 8:**
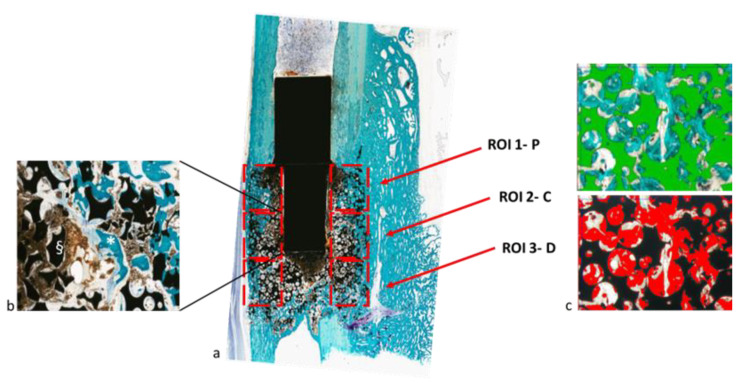
Representation of the regions of interests (ROIs) proximal (P), central (C) and distal (D) identified for histomorphometrical measures, 2x magnification (**a**). Representative high magnification of a ROI in which bone (*) and material (§) are marked (**b**). Snapshot of the Leica Qwin software showing the binarization process for image segmentation to identify the material (in green) and the bone (in red) (**c**).

Histomorphometric bone measurements were performed in three consecutive regions of interest (ROIs) of 2588 × 1960 pixels digitalized at 4× magnification, by using the implanted magnet as a reference on both sides, progressively from the most proximal to the most distal portion, to assess bone regeneration. (Figure 8).

Images were acquired using an optic microscope (Olympus BX51, Olympus Life Science, Waltham, MA, USA) equipped with an image analyzer system (Qwin, Leica Imaging Systems, London, UK).

To evaluate the scaffold healing process and osteointegration, the following static measurements were performed [25]:

- “Bone-to-implant” contact—BIC (%): the amount of bone contact at the interface level, defined as the percentage of implant perimeter with a direct “bone to implant” contact without intervening soft-tissue layers.
- New bone growth inside—B.Ar/T.Ar (%): the amount of new bone growth area (bone area) next to the scaffold in an area inside the scaffold (total area), expressed as a percentage.

### 4.6. AFM Analysis

An atomic force microscope (AFM) (NT-MDT, Moscow, Russia) for force spectroscopy (extraction of force–distance, F–d curves) was used. The cantilever stiffness was estimated using a thermal tuning method via NOVA software v. 1.1.1.19826- 2010 (NT-MDT, Moscow, Russia) according to the procedure suggested by Sader [26], and calibrated onto a TG1 (NT-MDT, Moscow, Russia) calibration grating. For each region investigated, 20 × 20 µm^2^ large, a grid of 4 × 4 F–d curves was acquired in the contact mode of operation. The curves obtained were then averaged and converted into F–δ curves, where δ is the penetration depth of the tip into the sample. The Young’s modulus (E) was extracted at each region by using a well-known contact model [27], assuming material isotropy and homogeneity. For calculation, spherical tip geometry (curvature radius R = 10 ± 1 nm) was assumed, according to manufacturer’s data [28]. The curves were acquired within the scaffold at points located at a constant distance (5.0 mm) from the *z*-axis, as sketched in Figure 6.

### 4.7. Statistical Analysis

A statistical analysis was carried out using software R and related packages (v4.1.2; R Core Team 2021). After having verified a normal distribution of data (Shapiro–Wilks test) and their homogeneity of variance (Levene test), a two-way ANOVA test was used to compare histomorphometric results by considering both treatment (2 levels: magnetic scaffold or magnetic scaffold + VEGF-MNPs) and region of interest (ROI, 3 levels: proximal, central and distal) as fixed factors. Post hoc pairwise comparisons were performed by adjusting *p* values according to Sidak–Holm.

## 5. Conclusions

The results of the present proof-of-concept study indicate that the creation of a “magnetic interface” enhances the bone regeneration for the fusion of a host bone with a magnetized scaffold. This trend is strongly mediated by the magnetic field which controls the distribution of the NP/VEGF aggregates and, hence, the bone regeneration.

## Data Availability

The data presented in this study are available in “Materials and Methods” and “Results” sections.
